# The effect of moderate intensity exercise in the postprandial period on the inflammatory response to a high-fat meal: an experimental study

**DOI:** 10.1186/s12937-016-0134-4

**Published:** 2016-03-08

**Authors:** Colby S. Teeman, Stephanie P. Kurti, Brooke J. Cull, Sam R. Emerson, Mark D. Haub, Sara K. Rosenkranz

**Affiliations:** 1 Department of Food, Nutrition, Dietetics and Health, Kansas State University, 212 Justin Hall, 1324 Lovers Lane, Manhattan, KS 66506 USA; 2 Department of Kinesiology, Kansas State University, 1A Natatorium, 920 Denison Ave, Manhattan, KS 66506 USA; 3 Physical Activity and Nutrition-Clinical Research Consortium (PAN-CRC), Department of Human Nutrition, Kansas State University, 1105 Sunset Ave, Manhattan, KS 66502 USA

**Keywords:** High-fat meal, Aerobic exercise, Postprandial lipemia, Inflammation

## Abstract

**Background:**

Consuming a high-fat meal (HFM) may lead to postprandial lipemia (PPL) and inflammation. Postprandial exercise has been shown to effectively attenuate PPL. However, little is known about the impact of postprandial exercise on systemic inflammation and whether PPL and inflammation are associated. The purpose of this study was to determine whether moderate intensity exercise performed 60 min following a true-to-life HFM would attenuate PPL and inflammation.

**Methods:**

Thirty-nine young adults (18–40 year) with no known metabolic disease were randomized to either a control group (CON) who remained sedentary during the postprandial period or an exercise (EX) group who walked at 60 % VO_2peak_ to expend ≈ 5 kcal/kgbw one-hour following the HFM. Participants consumed a HFM of 10 kcal/kgbw and blood draws were performed immediately before, 2 h and 4 h post-HFM.

**Results:**

At baseline, there were no differences between EX and CON groups for any metabolic or inflammatory markers (*p* > 0.05). Postprandial triglycerides (TRG) increased from baseline to 4 h in the EX and CON groups (*p* < 0.001), with no differences between groups (*p* = 0.871). High density lipoprotein cholesterol (HDL-C) decreased in both groups across time (*p* < 0.001) with no differences between groups (*p* = 0.137). Interleukin-6 (IL-6) was significant as a quadratic function over time (*p* = 0.005), decreasing from baseline to 2 h then increasing and returning to baseline at 4 h in all participants with no difference between groups (*p* = 0.276). Tumor necrosis factor-alpha (TNF-α) was not different from baseline to 4 h between groups (*p* > 0.05). There was an increase in soluble vascular adhesion molecule (sVCAM-1) from baseline to 4 h (*p* = 0.027) for all participants along with a group x time interaction (*p* = 0.020). Changes in TRG were associated with changes in interleukin-10 (IL-10) from 0 to 2 h (*p* = 0.007), but were not associated with changes in any other inflammatory marker in the postprandial period (*p* > 0.05).

**Conclusions:**

Despite significant increases in PPL following a HFM, moderate intensity exercise in the postprandial period did not mitigate the PPL nor the inflammatory response to the HFM. These results indicate that in populations with low metabolic risk, PPL and inflammation following a HFM may not be directly related.

## Background

A typical Western lifestyle allows many individuals to spend a significant portion, if not the majority of their day in a postprandial state [1]. The Western diet is typically calorically dense and nutrient poor, and consumption of this type of diet is associated with high levels of circulating triglycerides (postprandial lipemia (PPL)), which is an established independent risk factor for cardiovascular disease (CVD) [2]. The PPL response for most Western style meals that contain between 20 and 40 g of fat, is directly proportional to the amount of fat ingested in the meal [3].

Elevations in postprandial triglycerides have been shown to reduce endothelial function [4] and increase inflammatory biomarkers [5]. As with PPL, chronically elevated levels of systemic inflammation are associated with CVD and cardiac events [6, 7]. Aerobic exercise has been shown to effectively reduce lipemia in both the fasted and postprandial states, and to have both acute [8] and chronic [9] anti-inflammatory properties. As CVD continues to be a prominent cause of mortality in Western society, the relationship between PPL, exercise, and postprandial inflammation is of great interest. However, most studies examining exercise, PPL, and inflammation have used meal and/or exercise conditions that are not representative of the typical day-to-day lifestyles of modern Western Societies.

Exercise has been shown to be an effective means of attenuating PPL when performed both pre and post-meal, and at varying intensities. Moderate intensity exercise performed prior to the ingestion of a single high-fat meal (HFM) has been consistently shown to attenuate PPL [10]. However, many studies have not tightly controlled for overall energy balance between the bout of exercise and the HFM the next day. When caloric replacement has been allowed, the attenuating effects of the exercise on PPL have been greatly diminished [11]. Similar to moderate intensity pre-meal exercise, low intensity exercise performed before a meal, may also effectively attenuate PPL at relatively low energy expenditures (300–500 kcal). This is usually achieved in sedentary individuals without caloric replacement [12]. While not studied as frequently as pre-meal exercise, post-meal exercise has also been shown to be similarly effective at attenuating PPL when performed at a moderate intensity. However, previous studies have used exercise bouts of ≈ 90 min with large energy expenditure [13, 14]. Currently, little is known regarding the efficacy of shorter bouts of exercise, with energy expenditures that could be achieved during a typical work day, for attenuation of PPL and inflammation when exercise is performed following a HFM.

It has been demonstrated on several occasions that a single HFM can result not only in elevated lipids, but also in a pro-inflammatory response [15, 16]. Researchers have speculated that an exaggerated PPL response following a high-fat, high-energy meal may lead to an inflammatory response. Prolonged elevation of postprandial triglycerides may lower HDL cholesterol [17] and increase concentrations of small, dense, atherogenic, LDL cholesterol and chylomicron remnants [18]. However, it has been shown that prior exercise may be effective at lowering triglycerides while having no effect on the inflammatory response to a HFM [19, 20]. Given that both of these prior studies used a bout of exercise that was completed the day before ingestion of the HFM, and therefore far in advance of the HFM, potential anti-inflammatory benefits of the acute exercise may not have been observable. Currently, little is known about the relationship between exercise, PPL, and inflammation when a feasible bout of moderate exercise is performed after the ingestion of a HFM of regularly consumed proportions.

Therefore, the purpose of the current study was to determine whether or not a true-to-life bout of moderate intensity exercise in the postprandial period would attenuate the lipemic and inflammatory responses following a true-to-life HFM. Additionally, we examined whether there were associations between the changes in lipemia and changes in inflammation in the postprandial period. We hypothesized that a moderate intensity bout of exercise in the postprandial period as compared to remaining sedentary, would result in an attenuated PPL and inflammatory response to a HFM. Furthermore, we anticipated the attenuation in PPL would be associated with an attenuation in inflammation in the postprandial period.

## Methods

### Participants

Forty college-aged participants were recruited and thirty-nine completed this study (age 25.1 ± 5.4 years; 19 M 20 F). One participant was unable to complete the study due to failure to complete the meal in the allotted time. All participants were healthy, non-smokers, free of cardiovascular or metabolic disorders as assessed by a medical history questionnaire. All participants had fewer than two CVD risk factors according to CVD risk factor thresholds [21] and were not on medication to control asthma, blood pressure, blood glucose, or weight. To be eligible to participate, participants had to: 1) meet physical activity guidelines (≥150 min of moderate-to-vigorous physical activity (MVPA) per week) [22] or 2) participate in < 30 min of MVPA per week as measured by a short-form International Physical Activity Questionnaire (IPAQ). Written and verbal consent were obtained from all participants. The study was approved by the Institutional Review Board Involving Human Subjects at Kansas State University IRB#6622, and conformed to the Declaration of Helsinki.

### Experimental design

Participants visited the laboratory on two separate occasions with eight to twenty-one days between visits. During the first visit, participants completed medical history and physical activity questionnaires followed by anthropometric measurements including height, weight, waist circumference, and body fat percentage. Participants then completed a treadmill incremental test to exhaustion to determine peak aerobic capacity (VO_2peak_). VO_2peak_ was also used to calculate the duration of exercise to be performed on the second visit day for those randomized to the exercise (EX) group. Block randomization by a random number generator with a 1:1 group allocation occurred after participants signed up for the study, but was not revealed to them until after the exercise test. Participants were randomized to either the control (CON) condition, in which the participants were asked to remain sedentary throughout the postprandial period, or the EX condition, in which the participants performed a moderate intensity walk at 60 % of their VO_2peak_ during the postprandial period for a duration that would require them to expend half of the calories consumed from the HFM. All participants kept a detailed 3-day food log prior to their second laboratory visit. Participants were given a standardized, prepackaged frozen meal and allowed to self-select additional commercially available items that included a fruit cup, snack crackers, and snack cakes the day before their second laboratory visit. They were asked to consume the meal twelve hours before their appointment and then remain fasted until their arrival at the laboratory. Participants were asked to document any part of the standard meal they did not consume, and abstain from caffeine, alcohol, and exercise for at least 24 h prior to their second visit.

**Fig. 1:**
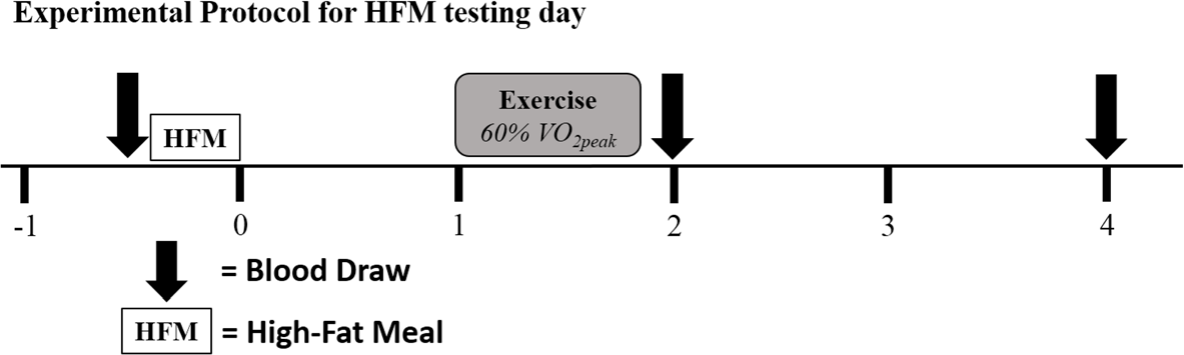
Timeline for participants’ second visit to the laboratory. Exercise occurred only in participants randomized to the EX condition while those in the CON condition remained sedentary for the duration of the postprandial period

The timeline for the second laboratory visit is shown in Fig. 1. Following the 12-h fast, participants arrived between 6:00–10:00 am to begin data collection. A catheter was inserted via antecubital vein and blood samples were drawn for triglycerides, glucose, and cholesterol assessments. After these measurements were complete, the remaining blood sample was centrifuged and plasma samples were pipetted into cryovials and stored at −60° C. Following baseline blood-sampling procedures, participants were allowed 20 min to consume the HFM (described below). Additional blood draws were performed at 2 and 4 h post-HFM.

### Tests and measurements

#### Anthropometric measures

All anthropometric measurements were completed by a trained research assistant using standardized methods in accordance with previous work from our laboratory [23]. Height, weight, and waist circumference were each measured twice, and a third measurement was taken if the recorded values differed by more than 0.5 cm or 0.5 kg. The two measurements within the acceptable difference range were averaged and this value was used for analyses. Height was measured with a SECA 214 portable stadiometer (Invictus Plastics, Leicester, England) and weight was measured with a digital scale (Pelstar LLC, Alsip, IL, USA). Waist circumference was measured with Gulick spring loaded measuring tape in the horizontal plane of the iliac crest (Accufitness, Greenwood Village, CO, USA). Body fat percentage was assessed via dual energy X-ray absorptiometry (DEXA) scan (GE Lunar Prodigy, Madison, WI, USA).

#### Questionnaires

Participants also completed a medical history questionnaire to verify they were free of cardiovascular and metabolic disease and met all inclusion criteria. All participants completed a previously validated short-form IPAQ [24]. This questionnaire asks participants to recall their physical activity (PA) behavior for the last seven days. PA is categorized into vigorous, moderate, walking, and sedentary time. All participants noted how many of the prior seven days they had participated in each one of those physical activity categories, and how much time on average they spent in each behavior per day. The IPAQ was used to establish whether participants met the PA inclusion criteria as previously described.

A 3-day food log (2 weekdays and 1 weekend day) was completed before the second laboratory visit to determine total dietary calorie and macronutrient distribution. Upon arrival for their second visit, participants reviewed their diet records with a trained research assistant who subsequently entered the dietary information into computer software to eliminate any confusion or possible mistakes on the entries. The 3-day food record was analyzed using Nutritionist Pro nutrient analysis software version 5.2.0 (Axxya Systems-Nutritionist Pro, Stafford, TX).

#### Peak aerobic capacity

An incremental exercise test to exhaustion on a treadmill (Precor 932i, Woodinville, WA, USA) was performed to assess peak aerobic capacity. Heart rate was measured using a chest strap heart rate monitor (Polar Wear Link Coded, Polar Electro Inc, Lake Success, New York). Substrate oxidation was assessed via breath-by-breath analysis of O_2_ consumption and CO_2_ production (Parvomedics TrueOne 2400 Metabolic Cart, Sandy Utah). The Borg scale rating of perceived exertion (RPE) from 0 to 20 was used to assess perceived effort at the end of each stage. The incremental protocol began at a participants’ fastest self-perceived 5 km walk/run pace at a 2 % incline. Speed was increased by 0.5 mph every two minutes, and beginning at the fourth stage (6 min into the test), speed was increased by 0.5 mph and incline was increased by 1 % at the beginning of each stage. Heart rate and VO_2_ were continuously reported and RPE was recorded 30 s prior to the end of each stage. Successful test criteria included: heart rate within ±1SD of the participant’s age predicted maximal heart rate (220-age), respiratory exchange ratio > 1.10, or if the participant was unable to continue. VO_2peak_ was recorded as the highest 15-s average measurement over the duration of the test.

#### High-fat meal

The HFM consisted of 10 kcal per kg of body weight (Jimmy Dean’s Meat Lovers Breakfast Bowl™) and 63 % of calories were from fat. Participants were required to ingest the entire meal within 20 min of their first bite. The nutritional composition of the meal was 460 cal per bowl, consisting of 33 g of fat, 265 mg of cholesterol, 17 g of carbohydrate, and 24 g of protein. Total kilocalories consumed ranged from 450 to 1113, with fat content ranging from 32.2 to 79.8 g. One participant was unable to complete the meal within the allotted time, citing intense dislike for the taste and texture of the provided meal.

#### Blood samples

Blood samples were taken at baseline prior to consumption of the HFM, and at 2 h and 4 h hours after the HFM. Blood draws were completed by first removing saline from the catheter line with a 3 ml syringe and then drawing the whole blood sample through a 5 ml syringe (BD, Franklin Lakes, NJ, USA). The whole blood samples were deposited into a 6 mL K2 EDTA BD Vacutainer (BD, Franklin Lakes, NJ, USA). Alere Cholestech LDX Lithium heparin capillary tubes were used to draw blood from the vacuum tubes for lipid analysis (Cholestech LDX Analyzer, Alere San Diego Inc., San Diego, CA). The remaining blood in the vacuum tube was centrifuged by a CxR Centrifuge (LW Scientific, Lawrenceville, GA, USA) and plasma samples were immediately pipetted into cryovials (Fisher, Hanover Park, IL, USA) and stored at −60 °C. After all participants had completed the experimental protocol, plasma samples were shipped to Eve Technologies (Calgary, Alberta, Canada) to be analyzed. In this study we quantified seven biomarkers using three different custom-plex assays. A Human High Sensitivity Cytokine 5-Plex, a Human Neurodegenerative Panel 2 Single Plex and a Neurodegenerative Panel 3 Single Plex. The multiplex assays were performed according to the protocol at Eve Technologies by using the Bio-Plex™ 200 system (Bio-Rad Laboratories, Inc., Hercules, CA, USA). The cytokine 5-plex consisted of IL- 1β, IL-6, IL-8, IL-10, and TNF-α with assay sensitivities ranging from 0.11 to 0.48 pg/mL. The Neurodegenerative Panel 2 single plex consisted of CRP with assay sensitivity of 0.0022 ng/mL. The Neurodegenerative Panel 3 single plex consisted of the cellular adhesion molecules soluble intercellular adhesion molecule-1 (sICAM-1) and soluble vascular cell adhesion molecule-1 (sVCAM-1) with assay sensitivity being 6.44 pg/mL.

#### Post-HFM exercise

Participants randomized to the EX group were instructed to begin walking on the treadmill exactly 60 min after completion of their meal. Participants were pre-fitted with the same Polar Wear Link Coded chest strap heart rate monitor as used during their VO_2peak_ test. Exercise was performed within ± 5 bpm of their heart rate at 60 % VO_2peak_ under close supervision of a research assistant.

The calculation used for determining exercise duration was as follows:

$$ \frac{bwkg \times 10\  calories \times 50\%\kern0.37em  of\  calories\  consumed\ }{\left(\left(\left(\left(VO2 peak\div 1000\right)\times bwkg\right)\times \frac{4.825 kcal}{liters\  oxygen}\right)\times 60\%VO2 peak\right)} $$

### Statistics

Data analyses were conducted using IBM SPSS Statistics v22.0 (IBM Corporation, Armonk, NY). Pearson’s r and Spearman’s rho 2-tailed correlations were performed to assess associations between independent and dependent variables, as well as between PPL and inflammatory markers for parametric and non-parametric data respectively. AUC analysis was performed using GraphPad Prism 6 (GraphPad Software, Inc. La Jolla, CA, USA) and independent sample *t* tests were performed to compare AUC between conditions. AUC analyses were performed for the metabolic outcomes of glucose, TRG, and high density lipoprotein cholesterol (HDL-C). Comparisons between EX and CON groups were performed using a two-way analysis of variance where condition was the between-subjects factor and time (baseline, 2 h, and 4 h) was the within-subjects factor. If parametric assumptions were not met, data were transformed (Lg10) and subsequent tests were performed. For data where transformation could not correct normality (glucose, IL-8, sVCAM-1), EX and CON groups were compared using Friedman’s tests with Mann–Whitney U, appropriate non-parametric alternative tests for each time point. For all analyses, significance was set at *p* < 0.05.

## Results

### Participant characteristics

Participant characteristics are shown in Table 1. Thirty-nine participants (19 M, 20 F) completed the study. Three participants were not included in the analysis of blood draw variables due to catheter insertion difficulties. There were no differences between EX and CON groups for any anthropometric variables. Twenty-two subjects were classified as overweight (BMI > 24.9).

**Table 1:** Participant characteristics

		Condition	
	All participants	EX	CON
Sex	19 M; 20 F	9 M; 10 F	10 M; 10 F
Age (years)	25.1 ± 5.4 (18–38)	25.8 ± 5.9	24.5 ± 5.0
Height (cm)	171.1 ± 10.4 (152.9–193.5)	171.3 ± 10.4	171.0 ± 10.6
Weight (kg)	75.1 ± 16.7 (45.5–111.3)	74.4 ± 15.9	75.8 ± 17.8
BMI (kg/m^2^)	25.5 ± 5.1 (17.4–42.0)	25.4 ± 5.5	25.7 ± 4.8
Body Fat (%)	27.1 ± 12.6 (5.4–55.5)	28.3 ± 12.2	25.9 ± 13.2
VO_2peak_ (ml/kg/min)	47.1 ± 11.6 (24.4–68.9)	47.6 ± 12.7	46.7 ± 10.8

Data are presented as mean ± SD. Ranges are shown in parenthesis for all participants. There were no differences between groups for any participant characteristics (*p* > 0.05)

### Energy balance

Energy balance data are shown in Table 2. The average amount of energy consumed was 748.5 ± 168.8 kcals, with no difference between groups. The average exercise energy expended on the treadmill in the EX group was approximately half of the energy consumed in the meal (377.8 ± 79.6 kcals). Total energy balance at 4 h included the estimated energy expenditure at baseline extrapolated to the 4 h postprandial time point. The time the EX group spent exercising was not included in the equation and exercise energy expenditure was added to the calculation separately.

**Table 2:** Energy balance

		Condition	
	All participants	EX	CON
Energy consumed	748.5 ± 168.8 (450.0–1113.0)	743.6 ± 159.1	758.4 ± 178.3
Energy expended on treadmill (EX only)	185.9 ± 196.4 (0–518.0)	^a^371.8 ± 79.6	N/A
Resting energy expenditure (REE) baseline to 4 h	292.9 ± 77.1 (176.6 ± 482.3)	295.0 ± 71.6	290.8.84.2
Total energy expenditure (EX + REE)	478.8 ± 222.6 (176.6–962.9)	^a^666.8 ± 142.1	290.8 ± 84.2
Total energy balance at 4 h	269.7 ± 214.3 (18.1–644.7)	^a^76.8 ± 52.3	462.7 ± 114.4

All data presented as kcals; mean ± SD. REE (resting energy expenditure). ^a^Indicates significant differences between EX and CON groups (*p <* 0.05)

### Metabolic data

**Fig. 2:**
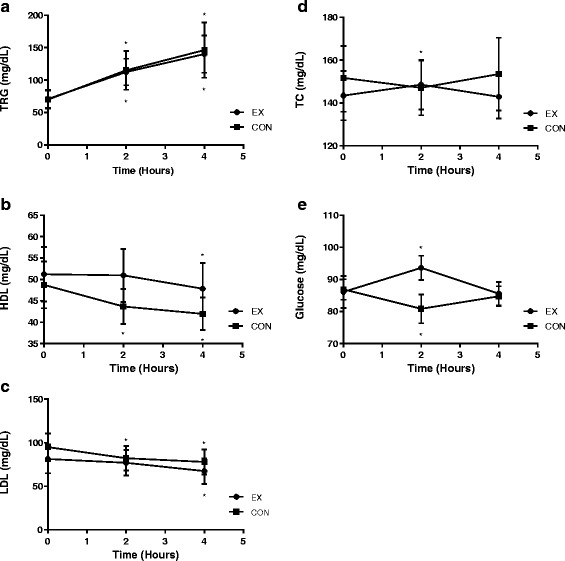
Metabolic responses to HFM between EX and CON groups at baseline, 2 h, and 4 h postprandially for (**a**) TRG (**b**) HDL (**c**) LDL (**d**) TC (**e**) Glucose. Circles indicate the EX group and squares indicated the CON group. *Indicates a pairwise change from baseline. Error bars indicate 95 % CI. Significance set at (*p <* 0.05)

**Fig. 3:**
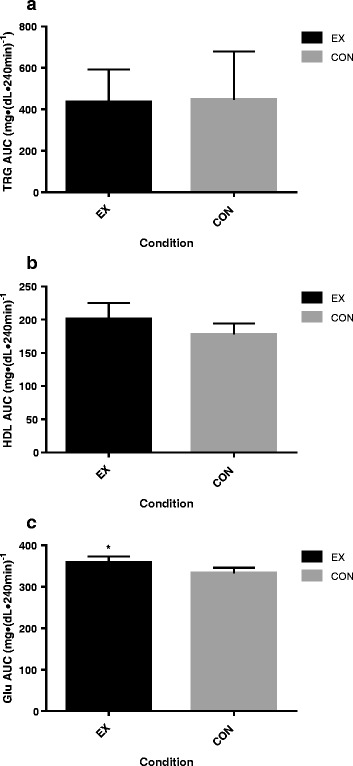
Metabolic total AUC responses to HFM between EX and CON groups at baseline, 2 h, and 4 h postprandially for (**a**) TRG (**b**) HDL (**c**) LDL. The black shaded area indicates the EX group and the grey shaded area indicates the CON group. * Indicates a difference between groups. Error bars indicated 95 % CI. Significance set at (*p* < 0.05)

Blood lipid and glucose data are presented in Table 3. There were no differences between the EX and CON groups at baseline for glucose, triglycerides (TRG), high-density lipoprotein cholesterol (HDL-C), low-density lipoprotein cholesterol (LDL-C), or total cholesterol (TC). Changes in metabolic markers over time are presented in Fig. 2 and total AUC data are presented in Fig. 3. TRG significantly increased from baseline to 4 h for all participants (*F* = 48.354, *p* < 0.001) with no differences between groups (*F* = 0.027, *p* = 0.871). Total TRG AUC was not different between groups (*p* = 0.871).

**Table 3:** Metabolic responses following a HFM

	EX	CON	Group effect	Time effect	Group x time
TRG (mg/dL)			*p* = 0.871		
Baseline	70.9 ± 29.2	69.8 ± 28.9			*p* = 0.807
2 h	112.4 ± 42.5^a^	115.1 ± 62.1^a^			
4 h	140.0 ± 59.4^a^	146.2 ± 88.3^a^		*p* < 0.001^c^	
HDL (mg/dL)			*p* = 0.137		
Baseline	51.2 ± 13.2	48.7 ± 11.3			*p* = 0.095
2 h	50.9 ± 12.9	43.7 ± 8.5^a^			
4 h	47.8 ± 12.4^a^	41.9 ± 7.8^a^		*p* < 0.001^c^	
^e^LDL (Lg10)			*p* = 0.471		
Baseline	1.90 ± 0.09	1.96 ± 0.14			*p* = 0.316
2 h	1.88 ± 0.09	1.89 ± 0.16^a^			
4 h	1.82 ± 0.11^a^	1.86 ± 0.17^a^		*p* < 0.001^c^	
TC (mg/dL)			*p* = 0.499		
Baseline	143.4 ± 23.9	151.7 ± 30.8			*p* = 0.023^d^
2 h	148.5 ± 24.1^a, b^	147.1 ± 26.6			
4 h	142.9 ± 21.1	153.5 ± 35.2		*p* = 0.961	
Glucose (mg/dL)			*p* = 0.071		
Baseline	86.1 ± 10.4	86.9 ± 6.7			*p* < 0.001^d^
2 h	93.6 ± 7.9^a,b^	80.8 ± 9.3^a^			
4 h	85.6 ± 7.6	84.7 ± 6.4		*p* = 0.277	

^a^Indicates a significant pairwise change from baseline

^b^Indicates a significant within subjects condition-by-time quadratic change

^c^Indicates a significant main effect

^d^Indicates a significant group x time interaction

^e^ Indicates data were Lg10 transformed

All data points met parametric assumptions except LDL and glucose at baseline. All data are presented as mean ± SD

Significance set at (*p <* 0.05)

HDL-C decreased from baseline to 4 h for all participants (*F* = 11.286, *p* < 0.001) with no group differences (*F* = 2.317, *p* = 0.137). Total HDL-C AUC was not different between groups (*p* = 0.103). LDL-C decreased from baseline to 4 h for all participants (*F* = 19.805, *p* < 0.001) and there were no differences between EX and CON groups (*F* = 0.537, *p* = 0.471).

For total cholesterol (TC) there was no significant main effect for group when comparing EX to CON (*F* = 0.466, *p* = 0.499). However, there was an overall group x time interaction (*F* = 3.962, *p* = 0.023). TC increased in the EX group from baseline to 2 h and then decreased from 2 h to 4 h and returning to baseline levels. Conversely, TC in the CON group decreased from baseline to 2 h before increasing and returning to baseline at 4 h.

There was no main effect for group between EX and CON (*F* = 3.842, *p* = 0.071) for glucose. However, total glucose AUC was significantly higher in the EX group (358.9 ± 29.6 mg/dLx240min)^−1^) compared to the CON group (333.3 ± 26.5 mg/dLx240min)^−1^) (*p* = 0.008). This difference was due to higher glucose concentrations in the EX group at the 2 h time point (*p* < 0.001). There was a significant group x time interaction for blood glucose (*F* = 16.150, *p* < 0.001). Glucose increased in the EX group at the 2 h time point before decreasing and returning to baseline at 4 h. In the CON group glucose decreased from baseline to 2 h before increasing and returning to baseline at 4 h.

### Inflammatory data

**Fig. 4:**
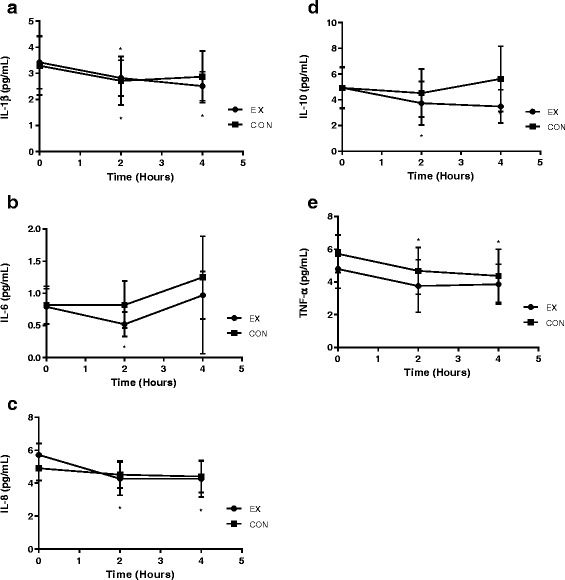
Inflammatory cytokine responses to HFM between EX and CON groups at baseline, 2 h, and 4 h postprandially for (**a**) IL-1β (**b**) IL-6 (**c**) IL-8 (**d**) IL-10 (**e**) TNF-α. Circles indicate the EX group and squares indicate the CON group. Circles indicate the EX group and squares indicate the CON group. *Indicates a pairwise change from baseline. Error bars indicate 95 % CI. Significance set at (*p <* 0.05)

Plasma markers of inflammation are presented in Table 4. There were no differences between EX and CON for any markers of inflammation at baseline (*p* > 0.05). Changes in markers of inflammation over time are presented in Fig. 4.

**Table 4:** Lg10 Inflammatory Responses following a HFM

	EX	CON	Group effect	Time effect	Group x time
hs-CRP (Lg10)					
Baseline	0.61 ± 0.69	0.69 ± 0.83			
2 h					
4 h					
IL-1β (Lg10)			*p* = 0.687		
Baseline	0.47 ± 0.19	0.44 ± 0.28			*p* = 0.727
2 h	0.39 ± 0.25^a^	0.35 ± 0.29^a^			
4 h	0.35 ± 0.22^a^	0.32 ± 0.38		*p =* 0.007^b^	
IL-6 (Lg10)			*p* = 0.276		
Baseline	−0.17 ± 0.24	−0.16 ± 0.26			*p* = 0.184
2 h	−0.39 ± 0.33^a^	−0.18 ± 0.28			
4 h	−0.11 ± 0.33	−0.06 ± 0.40		*p* = 0.012^b^	
^d^IL-8 (pg/ml)			*p* = 0.712		
Baseline	5.72 ± 1.27	4.90 ± 1.44			*p* = 0.311
2 h	4.27 ± 1.88^a^	4.52 ± 1.58			
4 h	4.27 ± 2.08^a^	4.40 ± 1.86		*p* = 0.036^b^	
IL-10 (Lg10)			*p* = 0.298		
Baseline	0.64 ± 0.22	0.62 ± 0.27			*p* = 0.212
2 h	0.45 ± 0.35^a^	0.56 ± 0.28			
4 h	0.46 ± 0.29^a^	0.62 ± 0.35		*p* = 0.094	
^d^TNF-α (pg/ml)			*p* = 0.310		
Baseline	4.79 ± 1.93	5.72 ± 1.89			*p* = 0.838
2 h	3.75 ± 2.66	4.68 ± 2.36^a^			
4 h	3.86 ± 2.02	4.37 ± 2.69^a^		*p* = 0.013^b^	
sICAM-1 (Lg10)			*p* = 0.746		
Baseline	1.89 ± 0.11	1.87 ± 0.10			*p* = 0.958
4 h	1.87 ± 0.15	1.86 ± 0.10		*p* = 0.370	
^d^sVCAM-1 (ng/ml)			*p* = 0.819		
Baseline	585.42 ± 125.58	599.30 ± 150.11			*p* = 0.020^c^
4 h	632.76 ± 134.96^a^	598.14 ± 141.08		*p* = 0.027^b^	

^a^Indicates a significant pairwise change from baseline

^b^Indicates a significant main effect

^c^Indicates a significant group x time interaction

^d^Indicates raw data that met parametric assumptions and did not require Lg10 transformation

hs-CRP was only measured at baseline, sICAM-1 and sVCAM-1 were only measured at baseline and 4 h. All data are presented as mean ± SD. Significance set at (*p <* 0.05)

Interleukin-1β (IL-1β) decreased from baseline to 4 h in all participants (*F* = 6.172, *p* = 0.007), with no differences between EX and CON groups (*F* = 0.165, *p* = 0.687). Interleukin-6 (IL-6) was significant as a quadratic function over time (*F* = 9.206, *p* = 0.005), decreasing from baseline to 2 h, then increasing and returning to baseline at 4 h in all participants with no difference between conditions (*F* = 1.231, *p* = 0.276). Interleukin-8 (IL-8) decreased from baseline to 4 h for all participants (F = 3.975, *p* = 0.036) with no differences between groups (*F* = 0.139, *p* = 0.712). There were no changes for Interleukin-10 (IL-10) from baseline to 4 h (*F* = 2.620, *p* = 0.094) for all participants with no differences between groups (*F* = 1.122, *p* = 0.298). Tumor necrosis factor-α (TNF-α) decreased from baseline to 4 h (*F* = 4.740, *p* = 0.013) with no differences between EX and CON groups (*F* = 1.077, *p* = 0.310).

**Fig. 5:**
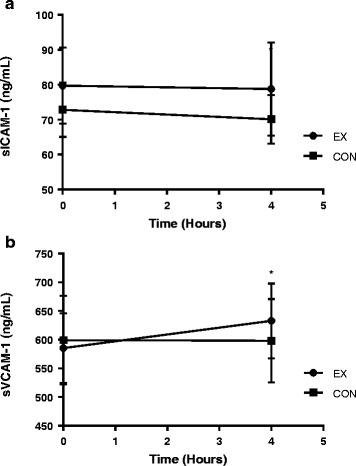
Cellular adhesion molecule responses to HFM between EX and CON groups at baseline and 4 h for (**a**) sICAM-1 (**b**) sVCAM-1. Circles indicate the EX group and squares indicate the CON group. * Indicates a pairwise change from baseline. Error bars indicate 95 % CI. Significance set at (*p <* 0.05)

Cellular adhesion molecules were measured at baseline and 4 h only, and these data are presented in Fig. 5. There were no differences between groups for soluble intercellular adhesion molecule-1 (sICAM-1) across time (*F* = 0.822, *p* = 0.370). There was an increase in soluble vascular adhesion molecule (sVCAM-1) from baseline to 4 h (*F* = 5.355, *p* = 0.027) for all participants along with a group x time interaction (*F* = 5.908, *p* = 0.020). The increase in sVCAM-1 was due to an increase in the EX group (*p* = 0.003).

### Associations between TRG and inflammatory markers

TRG was positively associated with TNF-α at baseline (*r* = 0.351, *p* = 0.038), but was not associated with IL-6, IL-8, IL-10, TNF-α, sICAM-1 or sVCAM-1 at any other time point (*p* > 0.05). Changes in TRG were associated with changes in IL-10 from 0 to 2 h (*r* = 0.467, *p* = 0.007), but no other associations were found between change in TRG and change in IL-6, IL-8, IL-10, TNF-α, sICAM-1 or sVCAM-1 from baseline to 2 h or 4 h postprandially (*p* > 0.05).

## Discussion

The purpose of this study was to investigate a more true-to-life approach to examining the impact of moderate intensity exercise on PPL and inflammation following a HFM. Our novel study design included walking at a moderate intensity for a duration of time needed to expend half of the calories consumed in a realistic HFM that was portioned relative to participant’s body weight. As anticipated, the HFM resulted in substantial PPL at both the 2 h and 4 h time points (≈100 % increase). In the current study, however, moderate intensity exercise in the postprandial period did not attenuate the postprandial rise in triglycerides as compared to CON, which disagrees with previous research. The lack of attenuation of triglycerides may be due to a relatively shorter bout of moderate intensity exercise that may not have been a sufficient stimulus to decrease hepatic VLDL secretion [25, 26]. Similarly, our hypothesis predicting lower inflammation in the EX group as compared to CON, was not supported. It has been previously shown that PPL is not always an accurate predictor of postprandial inflammation [19] and this was reinforced in the current study. Furthermore, there were no group differences between EX and CON when comparing changes in inflammatory markers over the course of the postprandial period, aside from the group x time interaction for sVCAM-1, which increased in the EX group as compared to CON.

The exercise duration in our study was intended to be representative of a more “doable” or true-to-life intensity and duration as compared to many previous studies. Where previous studies have demonstrated that moderate intensity postprandial exercise is effective for attenuation of PPL [13, 14], our findings showed no group differences between the EX and CON conditions with regard to PPL. A key difference between the current study and previous studies that have investigated postprandial exercise may be the lower exercise energy expenditure in the current study (~378 kcal) and absence of an overall energy deficit. Both of the aforementioned studies required participants to exercise at a light-to-moderate intensity for 90 min resulting in an energy expenditure greater than seen in the current study. In the study by Hardman & Aldred (1995), participants’ exercise resulted in an energy expenditure of ~585 kcal after ingestion of a meal of ~1178 kcal. Katsanos & Moffatt (2004) had participants exercise for an energy expenditure of ~829 kcal after a meal of ~1100 kcal. Although neither of these studies induced an energy deficit, their energy expenditures were much greater than those in the present study. Given the fact that our protocol did not induce an energy deficit, the absence of an attenuated PPL response in the EX condition in the current study is in agreement with a previous review that examined a possible threshold for energy deficit created via exercise performed the day prior to a meal. The authors concluded an exercise induced energy deficit of around 7 kcal/kgbw is required for aerobic exercise to effectively attenuate PPL [27]. It is possible that to effectively attenuate PPL, both exercise energy expenditure as well as overall energy balance from the day prior must be taken into consideration and should not be studied independently. More true-to-life energy expenditures, such as seen in our study, may not be enough of a stimulus to lower PPL in absence of an overall energy deficit.

Additionally, participants in the current study had metabolic responses that were highly heterogeneous, with increases in TRG ranging from 0 to 261 mg/dl from baseline to 4 h. Given that our participants also had highly varied aerobic capacities, we thought it was possible that the varied lipemic responses were due to the varied aerobic capacities and activity levels of our participants. Secondary data analyses revealed, however, that when VO_2peak_ was dichotomized into high and low groups, there were no between-group differences over time for PPL (data not shown). Nevertheless, in sedentary populations, individuals with lower aerobic capacities may require lower energy expenditures and shorter walking bouts to lower PPL as compared to more active individuals [12, 28]. Even though average triglycerides at 4 h were lower in the EX group compared to the CON group, high variability within both groups for all triglyceride time points effectively decreases the power to detect differences between groups. Post-hoc effect size was calculated for between-groups differences in TRG (*d* = 0.1568). This represents little to no effect on TRG for the EX condition.

Although exercise did not appear to effect postprandial triglycerides, the moderate intensity exercise in the present study did result in increased glucose AUC in the EX group as compared to the control. Increased glucose AUC in the EX group is likely a result of increased hepatic glucose production during the moderate bout of exercise. Decreased insulin secretion during exercise may stimulate increases in hepatic glucose production in an attempt to maintain glucose homeostasis during exercise [29]. Increased hepatic glucose production is likely due to increased glycogenolysis to provide additional energy substrate for exercising muscle [30]. Due to the relatively short time period between the completion of exercise and the 2 h blood sample in the current study, hepatic glycogenolysis may explain the higher glucose levels found in the EX group.

Reported values of many inflammatory measures in the postprandial period are inconsistent throughout relevant research literature. The main findings from our study were that there were no group x time interactions for inflammatory cytokines over time. Traditional pro-inflammatory cytokines did not change (IL-6), or they decreased across time (TNF-α, IL-8, IL-1β) with no group differences. IL-10, an anti-inflammatory cytokine, also decreased across time without differences between groups. Decreases in inflammatory markers seen in the current study may be due to participants in our study ingesting their HFM first thing in the morning. The diurnal variations of IL-6 and CRP have been shown to significantly decrease upon waking through the middle of the day [31]. The reduction of IL-6 in the current study may have led to a reduction of IL-10 [32] which would appear to represent increased inflammation. However, IL-10 reductions due to diurnal variations of IL-6 may not be representative of a true pro-inflammatory response to the HFM. The lack of inflammatory response to the meal may also be due to our young healthy participants being able to metabolically handle the HFM more easily than the larger, more unrealistic, meals seen in previous research.

Most previous literature has shown that inflammatory cellular adhesion molecules sICAM-1 and sVCAM-1 respond similarly in the postprandial period. However, our study only showed an increase in sVCAM-1 in the EX group. There was no change in sICAM-1 across time in either group. Changes in sVCAM-1 concentration immediately after exercise have been previously observed [33]. The lack of sICAM-1 response in the EX group may be due to an absence of muscle damage in our relatively short and moderate intensity bout of exercise [33]. In regard to the HFM, previous literature shows no increases in postprandial adhesion molecules in healthy subjects in most [34, 35], but not all [36] studies. Thus it appears that sVCAM-1 may be increased due to the bout of exercise and not the HFM.

In the EX group, IL-6 decreased from baseline to 2 h while in the CON group there was no change. Overall, IL-6 performed as a quadratic function over time, and the decrease in IL-6 from baseline to 2 h before returning to baseline at 4 h is a pattern that has been seen in previous literature. Lundman et al. [37] found IL-6 to be decreased 2 h postprandially before rising above baseline values at 4 h, and 6 h. Miglio et al. [16] found IL-6 to be elevated at all postprandial time points. However, both of these studies used meals of much higher energy intakes that used in our study. Lower IL-6 in the EX group at 2 h may support an acute anti-inflammatory effect of exercise as discussed by Petersen & Pedersen (2005) [8] or a diurnal variation as previously mentioned [27].

Although previous HFM research has produced mixed data for inflammatory markers, the decreases in TNF-α, IL-1β, and IL-8 over time in our study are in agreement with some previous research. TNF-α has been previously shown to have no change [38] or slightly decrease [39] across several time points after a HFM. There were no differences between groups for TNF-α over time and it does not appear to have been influenced by exercise. IL-1β decreased across time with no differences between groups. In healthy subjects, such as in the present study, IL-1β has previously been reported not to change in response to a HFM [40]. Since all of our participants were metabolically healthy and consumed a moderately portioned HFM, it can be reasonably expected that IL-1β would not increase, or possibly decrease as seen in our study. In the present study, IL-8 was also found to decrease over time with no difference between groups. IL-8 concentrations in the current study were similar to previous studies that provided meals of similar energy intake. Esser et al. [15] used a meal of ≈ 950 kcals and found IL-8 to increase postprandially, but the same study found a meal of more moderate energy intake (400 kcal) had no effect on IL-8. Myhrstad et al. [41] gave participants a meal of ≈ 700 kcal and also found no change in IL-8 concentrations. Considered together, the inflammatory markers used in the current study suggest the HFM given to our participants did not result in a pro-inflammatory state in the postprandial period.

Previous research has indicated that increased postprandial TRG and inflammation are linked. Our study, however, has shown a possible disconnect between the increase in postprandial TRG and increased inflammation. TRG were associated with TNF-α at baseline, but not with any other marker of inflammation at any time point. Despite the substantial rise in TRG at both 2 h and 4 h postprandially, our participants showed little sign of a pro-inflammatory state during the postprandial period. In agreement with Brandauer et al. [19] and previous work in our lab group [42–44] the current study demonstrates that the postprandial inflammatory process may be initiated by several factors (e.g. body composition, training status) other than increases in postprandial TRG alone.

A major strength of this study was the “true-to-life” approach to the HFM and postprandial exercise bout. Most previous studies have either used meals that exceeded the energy intake typically consumed in one sitting, and/or used a standardized meal given to participants regardless of body weight. The meal in the current study consisted of 10 kcal/kgbw (751.0 ± 167.4 kcal). This meal size would be more typical of an amount that an individual may eat for breakfast on a typical day. This meal allowed us to measure postprandial inflammation under similar metabolic conditions as would be seen on a day-to-day basis. In accordance with the “true-to-life” approach to the test meal, participants were allowed one hour to digest their meal before the EX group performed a moderate intensity walk that lasted ~39 min and expended ~372 kcal of energy. This bout of exercise represented an intensity, duration, and energy expenditure that many individuals would be able to regularly complete, particularly in the postprandial period, and potentially within the structure of a typical work-day.

A potential limitation to this study was only measuring blood lipids and inflammatory cytokines at two postprandial time points and cellular adhesion molecules at one postprandial time point. The postprandial response to blood lipids and inflammatory markers is very complex and highly variable among participants. Many previous studies have measured blood lipid and inflammatory markers at a greater number of time points over a longer period of time. The design of our study may have resulted in missed potential changes in blood and plasma analytes that could have been seen at the one hour or three hour postprandial time points. Additional changes could have also occurred at time points beyond four hours and these would have not been measured in our study.

The participants in the current study were young adults with no known metabolic diseases, many of whom were highly active and had higher aerobic capacities than an average individual. However, there were some overweight and obese participants included in the current study due to difficulties recruiting normal weight individuals who participated in no planned physical activity. All participants, regardless of BMI, had fewer than two CVD risk factors. Although the development of atherosclerosis has been shown to occur early on in life, evidence of this slowly developing inflammatory disease may not be shown by the postprandial response to a single meal in young healthy subjects, particularly with caloric and fat intakes which are more typical for a breakfast that would be consumed by an adult. Even though the meal given to our participants was high in fat (63 % fat) and resulted in a large increase in postprandial triglycerides, the moderate overall caloric content of the meal may explain the absence of postprandial systemic inflammation.

## Conclusion

The findings of the current study suggest that a single HFM, when portioned to the size of a typical breakfast, does not induce an inflammatory response. The evidence to support HFM induced inflammation has been well established, but our study suggested that among individuals with low metabolic risk, an unusually large energy and fat containing meal may be required to create this inflammatory state. Future studies should focus on populations with higher metabolic risk (advanced age, obese, abnormal blood lipids, sedentary, etc.) to determine whether a true-to-life HFM is enough of a stimulus to induce postprandial inflammation. Overall, this study demonstrates that following a true-to-life breakfast, compared to remaining sedentary, a moderate-intensity bout of exercise in the postprandial period, did not attenuate PPL or inflammation.

## Abbreviations

PPL: postprandial lipemia
HFM: high-fat meal
TRG: triglycerides
CVD: cardiovascular disease
IL-1β: interleukin-1 beta
IL-6: interleukin-6
IL-8: interleukin-8
IL-10: interleukin-10
TNF-α: tumor necrosis factor-alpha
CRP: C - reactive protein
sVCAM-1: soluble vascular cell adhesion molecule-1
sICAM-1: soluble intercellular adhesion molecule-1
